# The Network Structure of Personality Pathology in Adolescence With the 100-Item Personality Inventory for DSM-5 Short-Form (PID-5-SF)

**DOI:** 10.3389/fpsyg.2020.00823

**Published:** 2020-05-05

**Authors:** Amy Y. See, Theo A. Klimstra, Angélique O. J. Cramer, Jaap J. A. Denissen

**Affiliations:** ^1^Department of Developmental Psychology, Tilburg University, Tilburg, Netherlands; ^2^Department of Methodology and Statistics, Tilburg University, Tilburg, Netherlands

**Keywords:** personality disorders, adolescence, PID-5, network approach, personality pathology

## Abstract

There is currently a lack of understanding of the structure of personality disorder (PD) trait facets. The network approach may be useful in providing additional insights, uncovering the unique association of each PD trait facet with every other facet. A unique feature of network analysis is centrality, which indicates the importance of the role a trait facet plays in the context of other trait facets. Using data from 1,940 community Dutch adolescents, we applied network analysis to the 25 trait facets from the 100-item Personality Inventory for DSM-5 Short-Form (PID-5-SF) to explore their associations. We found that some trait facets only seem to be core indicators of their pre-ordained domains, whereas we observed that other trait facets were strongly associated with trait facets outside of their hypothesized domains. Importantly, anxiousness and callousness were identified as highly central facets, being uniquely associated with many other trait facets. Future longitudinal network studies could therefore further examine the possibility of anxiousness and callousness as risk marker trait facets among other PD trait facets.

## Introduction

There is hesitance in diagnosing personality disorders (PDs) in adolescence, as adolescent’s personality problems are viewed as transient and seen as being normative during this period that does not warrant clinical attention (Shiner and Allen, 2013). However, PDs are relatively common in adolescence, with estimated prevalence rates ranging from 10 to 15% (Shiner and Allen, 2013). In short, there is evidence indicating that PDs are common, stable, and non-normative during adolescence (Shiner and Allen, 2013). During adolescence, significant trait development occurs, and there is evidence suggesting that environmental and demographic changes during this age period may lead to the development of personality pathology features (e.g., Tackett et al., 2009). Therefore, it is important to examine the relation between PD features, which could potentially help our understanding of how to best assess PD in adolescents.

The most recent Diagnostic and Statistical Manual of Mental Disorders (DSM-5) (American Psychiatric Association, 2013) classifies individuals with specific PDs based on pre-defined combinations of symptoms. For example, a diagnosis of antisocial PD is given if three (or more) out of a set of seven symptoms are present. The categorical nature of this classification system has been criticized, as it obscures the significant heterogeneity among individuals diagnosed with the same PD (Trull and Durrett, 2005).

To address this issue (among others), the dimensional alternative model was introduced in Section III of the DSM-5, which empirically assesses trait facet levels (Krueger et al., 2012). The dimensional approach focuses on the degree to which a particular trait facet is present. More detailed information on each trait facet allows for the examination of specific relations among trait facets. Understanding the relations and differentiation among traits is of great relevance, since certain trait facets may be responsible for associations between pairs of other trait facets. Identifying such central trait facets could potentially advance knowledge on assessment and treatment in adolescent PD, for both research and clinical purposes.

Previous studies have investigated relations among trait facets using factor analysis, demonstrating that they can be integrated into five higher-order domains (Al-Dajani et al., 2016). In factor analysis, a domain is interpreted as responsible for the associations between trait facets (common cause). For example, the relations among eccentricity, perceptual dysregulation, and unusual beliefs could be attributed to a common factor – “psychoticism.” Overall, the factor analytical approach is useful in elucidating clusters of trait facets. However, previous research has shown that there are cross-loadings for certain trait facets, suggesting that not all relations among trait facets can be explained by higher-order factors. Therefore, further analyses are needed to fully understand the unique role that each trait facet plays, whereby each trait facet has a particular pattern of connections with other trait facets. To uncover the differential role of each trait facet, the network approach might provide useful additional insights.

Network analysis has been proposed as an alternative approach to studying the structure of personality (Cramer et al., 2012; Goekoop and Goekoop, 2014). For PD trait facets, which represent relatively stable between-persons differences (Johnson et al., 2000), the network approach could increase our understanding on the differential role of PD trait facets at the cross-sectional between-person level. According to this approach, trait facets may covary because of mutually reinforcing causal influences between facets, instead of the influence of a latent overarching construct. Because it is a less confirmatory approach and it is suitable for modeling complex associations between large numbers of attributes, the network approach could add on to findings from factor analyses by examining the unique role each trait facet plays, and more importantly the centrality of each PD trait facet.

Applied to personality pathology, the network approach estimates a network that consists of “nodes” that represent facets, and “edges” that represent associations between facets (Borsboom and Cramer, 2013). For example, callousness might be associated with hostility as well as impulsivity, and hostility might also be directly associated with impulsivity, resulting in an interconnected network. We used trait facets instead of items as nodes as self-report questionnaires may contain items that are similarly phrased, which would produce spurious nodes. The high number of items relative to the number of participants may also produce non-replicable edge estimates in the network (Costantini and Perugini, 2012). Moreover, when estimating central nodes, central trait facets would better reflect their more important role among other PD trait facet (cf. Hallquist et al. (2019).

Relative to factor analysis, the network approach provides additional information in several ways. First, the network approach introduces an important coefficient termed centrality, which can be applied in understanding personality pathology. Centrality indicates the importance of the role a trait facet plays in the context of other trait facets (Opsahl et al., 2010). For example, one could explore which trait facets play a more important role in Cleckley’s (1976) conceptualization of psychopathy. Cleckley’s work suggests that callousness, hostility, deceitfulness, manipulativeness, and grandiosity are trait facets belonging to psychopathy, and a network model would allow for identifying the most central among these facets. The identification of a central trait facet in a network of overall adolescent personality pathology may also be useful for clinical researchers who would like to empirically examine common tendencies that adolescents with personality pathology share.

Second, the network approach visualizes the relations among all trait facets. While we are aware that it is possible to examine the relations among all trait facets with factor analysis or by estimating a correlation matrix, the figures produced by network analysis software provide a very intuitive way to visualize associations (Bringmann and Eronen, 2018). These figures are much easier to interpret than a correlation matrix with 300 unique correlations among 25 facets.

Taken together, we investigated a network of associations between trait facets in a large sample of adolescents drawn from the general community. Before ascertaining whether trait facets can be used as nodes in a network, we first used confirmatory factor analysis (CFA) to ensure that the trait facets were separable. This approach reduces the chance that associations in the estimated network would emerge due to fuzzy construct boundaries. Exploring a network of a wide variety of PD trait facets is beneficial in helping us identify relations between trait facets and trait domains. Moreover, unique to network analysis, one can investigate which trait facets are more central in the network and thereby identify core features in the structure of personality pathology. Thus, the aim of the current study was to estimate a trait facets network in a large community adolescent sample, particularly focusing on identifying central trait facets in this network.

## Materials and Methods

### Participants and Procedure

We approached 2,190 adolescents attending various high schools in the south of The Netherlands. Of these, 1,940 adolescents (52.6% girls; Mage = 14.71 years, Age range = 10.48–17.62, *SD* = 0.77) agreed to participate. The data was part of the Project-Me study on identity problems and personality pathology. Parents and adolescents were informed that all information would be kept confidential and only utilized for research purposes. Passive consent was obtained from parents and students. Following approval from schools, data was collected in classrooms, with participants independently filling out the questionnaires on computers. Graduate students were present to guide and provide instructions. Participants did not receive incentives for their participation.

### Measure

The Personality Inventory for DSM-5 Short Form (PID-5-SF) is a 100-item abbreviated version of the original PID-5 that comprises of five higher-order domains (Antagonism, Detachment, Disinhibition, Negative Affectivity, and Psychoticism) and 25 lower-order trait facets (Krueger et al., 2012). Its internal structure replicated the structure of the original PID-5 in community and clinical samples (Maples et al., 2015; Bach et al., 2016). The translated items were taken from the Dutch version of the original PID-5 (De Clercq et al., 2014). Adolescents rated to what extent each item described themselves on a 4-point Likert scale ranging from 0 (very false or often false) to 3 (very true or often true). A decision was made to drop two items. One of the items (“The world would be better off if I were dead”) on the Depressivity facet was not included in the questionnaire based on directions from the Institutional Review Board (IRB) before approval was given, as it touched on suicidality. Another item on the Impulsivity trait facet (“I often do things on the spur of the moment”) was not included in the analyses because the item factor loading on the trait facet was low, probably due to the item being ambiguously phrased in Dutch. Mean scores, Cronbach’s Alpha and McDonald’s Omega (Deng and Chan, 2017) are reported in Table 1. Almost all trait facets had α ≥ 0.70 (18 out of 25) and ω ≥ 0.70 (21 out of 25).

**TABLE 1:** The 25 pathological personality facets and their corresponding labels, Mean (SD), Cronbach’s alpha, and McDonald’s Omega.

Label	Description	Mean (SD)	Cronbach’s alpha (α)	Mean inter-item correlations	McDonald’s Omega (ω)
**Facets (Domains)**					
AtS (A)	Attention Seeking	0.98 (0.61)	0.81	0.51	0.86
CaL (A)	Callousness	0.45 (0.52)	0.80	0.49	0.88
DFn (A)	Deceitfulness	0.63 (0.53)	0.70	0.37	0.81
GrD (A)	Grandiosity	0.40 (0.47)	0.74	0.42	0.82
Man (A)	Manipulativeness	0.84 (0.62)	0.79	0.49	0.90
AnH (DE)	Anhedonia	0.44 (0.47)	0.71	0.38	0.61
DeP (DE, NA)	Depressivity	0.38 (0.55)	0.77	0.55	0.97
InA (DE)	Intimacy Avoidance	0.90 (0.61)	0.71	0.39	0.76
Susp (DE, NA)	Suspiciousness	0.69 (0.49)	0.60	0.28	0.79
WiD (DE)	Withdrawal	0.52 (0.51)	0.63	0.29	0.85
Dis (DI)	Distractibility	1.47 (0.84)	0.89	0.68	0.94
Imp (DI)	Impulsivity	1.13 (0.62)	0.69	0.44	0.70
Ire (DI)	Irresponsibility	0.56 (0.47)	0.58	0.25	0.98
RPf (DI)	(lack of) Rigid Perfectionism	1.96 (0.64)	0.71	0.40	0.92
RTk (DI)	Risk Taking	1.05 (0.69)	0.83	0.55	0.72
AnX (NA)	Anxiousness	0.94 (0.69)	0.80	0.50	0.81
EmL (NA)	Emotional Lability	0.90 (0.68)	0.78	0.48	0.93
Hos (NA, A)	Hostility	1.02 (0.60)	0.71	0.38	0.72
Per (NA)	Perservation	1.08 (0.54)	0.63	0.30	0.72
RA (NA, DE)	Restricted Affectivity	1.03 (0.58)	0.64	0.31	0.68
SI (NA)	Separation Insecurity	1.13 (0.59)	0.61	0.29	0.80
Sub (NA)	Submissiveness	1.02 (0.59)	0.73	0.41	0.56
EcC (P)	Eccentricity	0.85 (0.73)	0.84	0.58	0.62
PD (P)	Perceptual Dysregulation	0.44 (0.49)	0.70	0.36	0.83
UB (P)	Unusual Beliefs and Experiences	0.66 (0.64)	0.71	0.38	0.92

*A = antagonism, DE = detachment, DI = disinhibition, NA = negative affectivity, P = psychoticism.*

### Statistical Analyses

The full dataset comprised of 1,940 participants. As the network comparison test (NCT) is unable to handle missing data, we removed 4 participants who did not provide complete data.

#### Confirmatory Factor Analyses

Confirmatory factor analyses on the item-to-facet structure were conducted in a number of steps. First, we examined the fit of the hypothesized item-to-facet structure (Krueger et al., 2012). Next, measurement invariance across gender of the PID-5-SF factor structure was inspected to ensure that the PID-5-SF items captured trait facets similarly for boys and girls. We followed Steenkamp and Baumgartner’s (1998) suggestions to link forms of invariance to the purpose of the study. Because a network is based on associations between variables, it sufficed to test for configural and metric invariance. We correlated residuals between items belonging to the same facet. Details of the analyses are available in the Supplementary Material.

#### Network Analysis

All network analyses were conducted in R (R Development Core Team, 2014). Prior to estimating the network, non-paranormal transformation was applied to correct for non-normality in the marginal distribution of the data (Liu et al., 2009). Multivariate non-normality appears to be problematic only when testing one-factor versus multi-factor CFA models (Cain et al., 2017), and we did not run such comparisons. The network was estimated and visualized using the R-package “*bootnet*” (Epskamp et al., 2017). To estimate partial correlations in the network, a Gaussian graphical model (GGM) (Lauritzen and Wermuth, 1989) was fitted to the data. The estimated partial correlations network was undirected, with an absence of an edge between two trait facets indicating that they were not significantly correlated after controlling for the remaining trait facets in the network. To control for false positive correlations, we applied a least absolute shrinkage and selection operator (LASSO) procedure based on the Extended Bayesian Information criterion (EBIC) (Tibshirani, 2011; Costantini et al., 2015). Following Foygel and Drton (2010) suggestion, we set the LASSO tuning parameter to 0.5 to obtain a more parsimonious network that errs on the side of caution. Such a setting will lead to a network with fewer edges, avoiding spurious edges but potentially missing some edges (i.e., higher specificity). Moreover, it is more likely that a network with less true connections (i.e., higher sensitivity) that is based on the true network structure and sample size is obtained. Overall, a network with LASSO-EBIC (tuning parameter 0.5) regularization works particularly well in retrieving the true network structure that also allows for easier interpretable networks. This procedure facilitates a balance between parsimony and goodness of fit of the estimated network model. Moreover, regularized partial correlations prevent overfitting of data in the presence of numerous covariates, which might otherwise lead to non-replicable results (McNeish, 2015). Partial correlations are preferred over zero-order correlations as the latter might result in spurious associations that arise from indirect associations via other trait facets. Under some conditions, partial correlations can produce unreliable results due to suppression effects. However, there is accumulating evidence for the replicability of these adjusted partial correlation-based network models (Costantini et al., 2015; Epskamp et al., 2016). Also, robustness checks of results can be conducted after estimating the network (Epskamp et al., 2017). For reasons of completeness, we estimated networks that were based on both partial and zero-order correlations.

The “nodes” of the partial correlations network represent the 25 PID-5-SF trait facets, and “edges” indicate regularized partial correlations between two trait facets. The strength of the association between two nodes is indicated by the thickness and saturation of the edge (Epskamp et al., 2012). We used an algorithm that places nodes with stronger average associations closer to the center of the network, and nodes with weaker average associations toward the periphery (Fruchterman and Reingold, 1991). Only edges with partial correlations ≥0.03 were included to aid visualization. For the zero-correlations network, an edge indicates a correlation between two trait facets. In this case, only edges with medium effect size (*r* > 0.30) were included for visualization.

Robustness analyses were conducted using the R-package “*bootnet*” (Epskamp et al., 2017). There are currently no clear guidelines on the minimum number of participants required per parameter (Fried and Cramer, 2017). Therefore, assessing the robustness of the estimated edge weights and centrality estimates is important. We performed several methods suggested by Epskamp et al. (2017). We estimated edge weights by drawing bootstrapped 95% confidence intervals (i.e., drawn randomly with replacement 1000 samples of the same size as the original sample, each time re-estimating the network). Robustness coefficients (randomly dropping 10, 20, …, 90% participants from the sample and recomputed centrality estimates) for centrality measures were generated to ascertain the replicability of the edge weights and the centrality measures. The Supplementary Material contains more information on these bootstrap procedures.

To test for differences in the estimated network across groups (i.e., boys versus girls, as well as random half splits of the total sample), network structure and global network strength were compared. Network structure refers to the structure of the network as a whole. Differences in network structure involves testing if this structure is invariant between groups. Global network strength is the weighted sum of the associations (absolute values) in the network. Differences in global network strength involve the difference in the overall sum of associations between groups. Network comparison tests (NCT) for network structure and global network strength were implemented using the R package “*NetworkComparisonTest*” (van Borkulo, 2016).

We computed the centrality of each node in the network with the following measures: strength, closeness, and betweenness (Opsahl et al., 2010). Strength centrality is calculated as the sum of the relevant edges (absolute values) of the connections of a specific node relative to all other nodes. A highly strength-central node has stronger direct connections with many other nodes. Closeness centrality is calculated as the inverse of the sum of the distances of a specific node from all the other nodes in the network. A highly closeness-central node has a short average distance to the remaining nodes in the network. Betweenness centrality is measured by calculating how often a specific node lies on the shortest path between all pairs of nodes. A high betweenness-centrality node is central in connecting other nodes, facilitating the flow of information through the network. The three measures of centrality were computed using the “*centralityPlot*” function in the “*q-graph*” package (Epskamp et al., 2012).

## Results

### Confirmatory Factor Analyses

The fit of a CFA model specifying the item-to-facet structure was close to acceptable. The item-to-facet model for girls and boys had a close to acceptable fit (CFI > 0.95, RMSEA < 0.06, and SRMR < 0.08) (Hu and Bentler, 1999). Thus, there was satisfactory configural invariance. Models with factor loadings constrained equal across girls and boys for the item-to-facet structure differed in terms of a significant χ^2^-difference test *(p* < 0.05), but ΔCFI and ΔRMSEA indices (ΔCFI < 0.010 and ΔRMSEA < 0.015) suggested that factor loadings were roughly equal for boys and girls, indicating metric invariance. Details of the CFA outputs and an explanation for correlating residuals are available in Supplementary Material.

### PID-5-SF Network and Robustness

**FIGURE 1:**
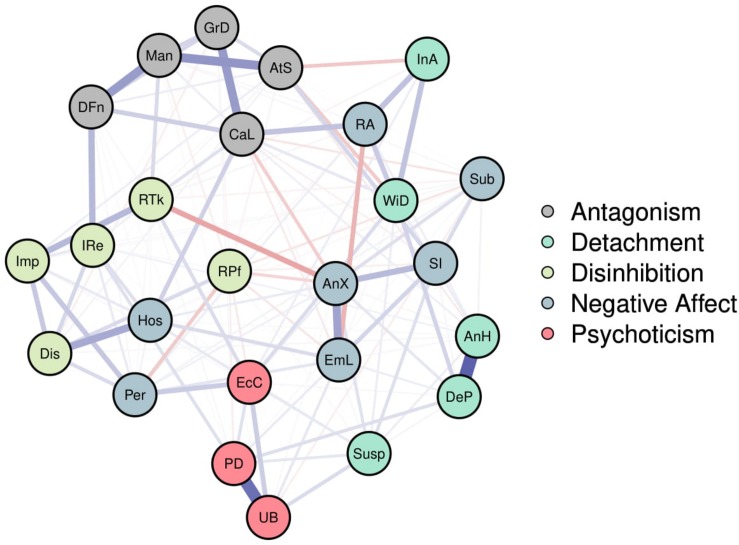
Network of the 25 PID-5-SF facets. Facets belonging to the same domain appear in the same color. Purple (solid) edges represent positive partial correlations between facets, while red (dotted) edges represent partial correlations.

Figure 1 visualizes the partial correlations network based on 25 PID-5-SF trait facets (see Supplementary Table S2 for the correlation matrix). In our settings, positive regularized partial correlations are colored blue (solid line) and negative regularized partial correlations are colored red (dashed line). Some trait facets seemed to be core indicators of their pre-ordained domains, whereas there were observable cross-loadings for others. Robustness checks provided evidence for the network being relatively well replicable across different subsamples. Detailed results of these checks are presented in the Supplementary Figures S1–S4. The NCT indicated that there were no significant differences in the PID-5-SF network structure across gender (*M* = 0.151, *p* = 0.09) or random half splits (*M* = 0.123, *p* = 0.237). The global network strength test similarly revealed no significant differences in the weighted sum associations of trait facets across gender (12.4 vs. 12.3; *p* = 0.84), nor across random half splits (12.8 vs. 12.1; *p* = 0.312) (see Supplementary Figures S5, S6).

### Associations of Trait Facets Within-Domains

To further inspect the associations between trait facets within the same domains, two aspects of these associations were examined. We first calculated the percentages of significant associations between trait facets within domains (see Table 2). This was defined as the percentage of the total number of possible associations found in the corresponding part of the network that were statistically significant. The percentages of within-domain associations between trait facets that were statistically significant was high (>80% of all possible associations, and even 100% for psychoticism), and generally higher than percentages of across-domain associations.

**TABLE 2:** The number (%) of PID-5-SF facets associations within and between domains.

	A	DE	DI	NA	P
A	12/15 (80%)				
DE	22/36 (61.1%)	12/15 (80%)			
DI	24/30 (80%)	17/30 (56.7%)	9/10 (90%)		
NA	34/53 (64.2%)	43/47 (91.5%)	29/45 (64.4%)	30/36 (83.3%)	
P	4/18 (22.2%)	13/18 (72.2%)	10/15 (66.7%)	12/27 (44.4%)	3/3 (100%)

*Within domain associations are highlighted in bold. The percentage of facet associations between and within domains (out of the maximum number of possible associations). The maximum number of associations within domains are calculated as follows: denoting TFx as the number of facets in domain x, the maximum number of associations within this domain is TFx(TFx-1)/2. The maximum number of associations between domains are generated by the product of the number of facets from one domain to the number of domain of the other domain. For both within- and between-domains, the number of associations found between facets were determined by inspecting the generated partial correlation matrix. The proportion of found associations over maximum number of within-/between-domains were taken to calculate percentages in the table.*

Two associations with medium effect sizes (*r* > 0.30) emerged between the following nodes: Anhedonia (AnH) and Depression (DeP) (*r* = *0.54*), and Perceptual Dysregulation (PD) and Unusual Beliefs and Experiences (UB) (*r* = *0.43*). Both associations involved trait facets belonging to the same domain.

### Associations of Trait Facets Across-Domains

The percentage of connections between the trait facets belonging to different domains was also generally high (Table 2). This was highest for facets of negative affectivity and detachment (i.e., 91.5% of all possible associations), followed by facets of antagonism and disinhibition (i.e., 80% of all possible associations). Therefore, trait facets that belonged to these domains tended to co-vary with facets of the corresponding other domains. The lowest percentage of cross-domain connections was found between trait facets belonging to the antagonism and psychoticism domains (i.e., only 22.2% of all possible associations).

### Centrality Estimates

**FIGURE 2:**
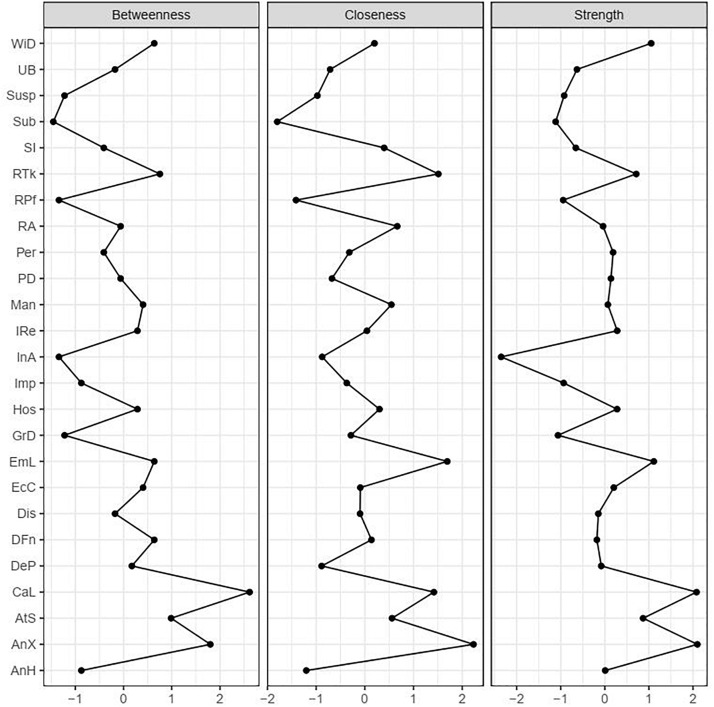
Standardized centrality estimates of the 25 PID-5-SF trait facets.

Strength, closeness, and betweenness centrality measures were computed across all 25 nodes (Figure 2). All centrality estimates were substantially interrelated (strength and closeness: *r* = 0.73; strength and betweenness: *r* = 0.88; closeness and betweenness: *r* = 0.82). Therefore, we followed previous studies (Armour et al., 2017; Santos et al., 2017) by limiting our interpretation of centrality to the most robust measure, which was strength centrality. The three nodes with the highest node strength centrality were (in descending order): Anxiousness, Callousness, and Withdrawal. The three nodes with the lowest node strength centrality were Intimacy Avoidance, Submissiveness, and Rigid Perfectionism.

## Discussion

The purpose of the current study was to explore the network structure of pathological personality trait facets based on the PID-5-SF in a large sample of community adolescents. To our knowledge, this is the first study investigating a variety of PD trait facets in such a sample. Unlike most previous network studies, we first established the separability of the network’s nodes. Factor analyses on the item-to-facet structure for the PID-5-SF supported this separability, as the hypothesized item-to-facet structure had an acceptable fit to the data. Next, we used the network approach to explore interconnections among these trait facets. The partial correlations network indicated that some trait facets only seemed to be core indicators of their pre-ordained domains, whereas other trait facets were associated with trait facets outside of their hypothesized domains. Therefore, while the PID-5 trait model holds and the facets predominantly belong to their predefined domains, the network also showed that almost all trait facets were directly or indirectly interconnected across domains. As such, personality pathology might be best described in terms of syndromes consisting of several pathological personality trait facets that are not necessarily restricted to one trait domain. In other words, we found that facets partly bridge domains, which is in agreement with current clinical practices in assessing PDs (e.g., Shiner and Allen, 2013). Importantly, unique to network analysis, we identified highly central facets, suggesting that these trait facets are common among different specific types of personality pathology.

### Associations of Trait Facets Within PID-5-SF Domains

Below we discuss in detail the findings relevant for further research using the network approach. The association between the facets Anhedonia and Depressivity was the strongest, followed by the association between the facets Perceptual Dysregulation and Unusual Beliefs and Experiences. Previous research on a psychiatric sample also reported that the association between trait Anhedonia and trait Depressivity was the strongest of all facet associations (Quilty et al., 2013). Similarly, and consistent with previous studies (Lewandowski et al., 2006), facets of unusual perceptual experiences and implausible beliefs were strongly associated. Therefore, our results replicated previous findings. These results, however, also need to be assessed in longitudinal studies to disentangle the processes through which Anhedonia affects Depressivity, and vice versa.

### Associations of Trait Facets Across PID-5-SF Domains

Some trait facets were associated with trait facets outside of their hypothesized domains, however. For example, there was a close clustering of trait facets belonging to the negative affectivity domain with those from the detachment domains, and the same was true for facets from the antagonism and disinhibition domains. This is consistent with previous research that found high co-occurrence of obsessive-compulsive PD and antisocial PD with borderline PD in outpatient adolescents (Becker et al., 2000; Loas et al., 2013), as these PDs have facets that belong to several domains (negativity, detachment, antagonism and disinhibition).

Generally, the relatively high number of associations across domains indicates that there are many facets that connect the different domains of dysfunctional personality. These facets might be compared to “bridge symptoms” (Borsboom and Cramer, 2013), which are assumed to play a role in linking personality pathology trait facets. Our findings showed that individuals with corresponding facets might face problems in multiple trait and/or PD domains. For instance, restricted affectivity (negative affectivity domain) was associated with callousness (antagonism domain) in our network. This indicates that of the adolescents exhibiting high negative affectivity, especially the ones with high levels of restricted affectivity, were likely to also report callousness-related tendencies.

The overall pattern of associations between PID-5-SF trait facets in adolescent is not often studied, and we reported some interesting findings. Relative to studying the overall pattern of associations from a correlation matrix, the figures produced by network analysis software provide a very intuitive way to visualize associations that is easier to interpret. However, from a data-analysis perspective, the main aspect that is unique to network analysis is the concept of centrality, which we will discuss below.

### Centrality

Some facets had a particularly high number of relatively strong associations with facets within and outside the same domain. Anxiousness was one such facet. Specifically, anxiousness had a negative association with risk-taking, and it seemed that this negative association provided a main bridge between trait facets linked to disinhibition and antagonism domains. Previous studies supported this negative association between anxiousness and risk-taking (Giorgetta et al., 2012; Yip and Côté, 2013). Possibly, an anxious person’s heightened vigilance to potential threats as well as pessimistic evaluation of future evens might be associated with reduced risk-taking behavior. Future longitudinal studies could further investigate the viable link between anxiousness and risk-taking among PD trait facets, as the bridge between the trait facets seem to be of potential importance for understanding comorbidity between several forms of personality pathology.

Callousness was also highly strength central. It is strongly connected with Hostility, Deceitfulness, Manipulativeness, and Grandiosity, which are all trait facets belonging to Cleckley’s (1976) conceptualization of psychopathy. This suggests that callousness could be the central risk factor among the other trait facets, or a common core. Future longitudinal studies could therefore investigate Callousness as a risk trait facet among other trait facets in the description of psychopathy.

Interestingly, Intimacy Avoidance had the lowest strength centrality, yet it was embedded in a cluster of facets with high Withdrawal and Restricted Affectivity and lowered Attention Seeking with rather strong associations. This suggests that Intimacy Avoidance has meaningful associations with a handful of other trait facets. Intimacy Avoidance is a rather specific facet, with elevated levels being indicative of an increased likelihood of also having elevated levels on a limited number of other facets. This is supported by previous research that intimacy avoidance stems from either a fear of intimacy or a lack of interest or motivation to become intimate with others, which has clear emotional and interpersonal ramifications (Bartholomew, 1990), which was reflected in the type of facets that co-occurred in the cluster. In comparison, despite having a slightly higher strength centrality, Suspiciousness seemed to have weaker individual relations with many other trait facets. Suspiciousness would thus appear to be a mild but more general indicator of maladaptiveness, but with rather weak associations with almost all other facets. As previously suggested, it is often unclear if Suspiciousness is more consistent with the notion that it is a consequence of extreme social anxiety, the result of the lack of close friends, or the reflection of psychotic-thinking (Kremen et al., 1998).

### Strengths and Limitations

The present study was characterized by several strengths. First, we had a large sample size – 1,940 participants – which is a strength and necessity for network models aiming to estimate a large number of parameters in a replicable way. In addition, we implemented recent recommendations following an extensive discussion on the replicability of networks (Borsboom et al., 2017; Forbes et al., 2017) by conducting extensive robustness checks. Specifically, we examined whether the network structure replicated in both gender groups and random split halves of the total sample. We also carried out a verification check of the partial correlations network, in which the zero-order correlation matrix generally concurred with our findings (see Supplementary Material Table S3). These steps increased confidence in the replicability of the estimated network structure, indicating that the findings were robust although corroboration in another sample is warranted. Second, we used facets instead of items in the network, potentially increasing the reliability of our findings. Despite these strengths, a number of limitations need to be taken into account when interpreting the results.

First, measuring pathological personality traits with only self-report data is limited, specifically due to self-representation and social desirability biases. Certain facets such as *unusual beliefs and experiences* and *cognitive and perceptual dysregulation* target highly subjective experiences that might be accurately captured with self-reports, but utilizing information from various informants (e.g., parents, teachers) might provide additional information. For example, such data would have allowed for examining whether results were replicable across raters, thereby potentially further validating the applicability of the PID-5-SF in adolescents and the network structure of personality pathology in this age group. Also, as suggested by Tackett (2010), multi-informant data is essential to establish construct validity of emerging personality pathology.

Second, two items were excluded from the analyses. One item was dropped as it touched on suicidality. Moreover, despite comprehensive Dutch translation of the measure that was approved by two of the authors of the original PID-5 article, one additional item had low item-factor loading on the impulsivity facet in our sample and had to be dropped as well. This could also have been due to our young sample. We acknowledge that the excluded items might have resulted in a loss of information, thereby changing (i.e., narrowing) the interpretation of their corresponding scales. However, because the PID-5-SF comprises a large number of items, it is unlikely that this would have greatly affected the reliability and validity of these scales. Also, in a separate study on Dutch undergraduate students (*n* = 218, 74.3% women), we correlated facet scores with and without the excluded items. These correlations were very high (depressivity with vs. without the excluded item: *r* = 0.98; impulsivity with vs. without the excluded item: *r* = 0.91). Therefore, the exclusion of the items likely did not substantially affect our findings.

Third, the current study estimated a cross-sectional network in an adolescent sample. Therefore, our results may not be generalizable to an adult population. However, studies have shown that the factor structure of the PID-5 was comparable between adolescents and adults (De Clercq et al., 2014; Fossati et al., 2015). This would suggest comparability as both factor models and the type of network model we used are based on between-person correlations. This comparability would still need to be tested in a network study on an adult sample drawn from a context comparable to the one that our adolescent sample was drawn from. Fourth, our findings may be exemplary of a group of individuals (between-person associations), and we can conclude that individual differences in a facet were associated with individual differences in another facet. These results do not warrant extrapolation to the level of the individual (within-person associations) (Molenaar and Campbell, 2009). As a result, we are not able to draw conclusions on causality or direction of effects from one facet to the other. Future studies might benefit by examining these associations at the within-person level, by employing longitudinal (e.g., experiencing sampling) methods. Alternatively, future studies could examine whether elevated scores on these facets are key risk factors in the development of PDs at a later age (Boschloo et al., 2015). While dynamic networks and cross-sectional networks provide different perspectives, cross-sectional networks provide important insights about between-person associations across various facets. Future studies utilizing longitudinal data could extend our study and further uncover the dynamic associations between behaviors indicative of pathological personality traits.

Finally, we did not assess clinical samples. Therefore, these findings are not generalizable to adolescents with diagnosed PDs. There is substantial evidence showing that pathological personality traits and normal levels of personality lie on the same continuum (Costa and McCrae, 2010). Of note, some adolescents in our sample were possibly close to clinical levels, as 190 adolescents who scored on average the highest (top 10%) across all 25 PID-5-SF facets, had overall facets mean values (*M* = 1.40) that were even slightly higher than those reported in studies examining patients with PDs (*M* = 1.03, *SD* = 0.67) (e.g., Wright et al., 2015). Therefore, our sample potentially covered the whole range of pathological to non-pathological personality, suggesting that our findings may have clinical relevance. However, future studies would benefit from such cross-sample validations in order to draw firm conclusions with clinical implications.

## Conclusion

The present study showed the differential roles each trait facet played in a trait facets network. We explored centrality, a unique feature of network analysis, and found anxiousness and callousness to be central trait facets in the network. Also, we found significant relations among trait facets within and across domains. Overall, these findings found important trait facet-to-trait facet associations that should be further explored with future longitudinal analysis of trait facet networks. Our findings could potentially guide future studies in unraveling the nature of subclinical personality pathology.

## Data Availability Statement

The datasets generated for this study will not be made publicly available as this was indicated in the informed consent. Requests to access the datasets should be directed to the corresponding author.

## Ethics Statement

As the study involved human participants, ethical approval was first obtained from the local IRB (EC-2015.49). Verbal passive consent was obtained from the participants prior to them completing the questionnaires. Passive consent was also provided by the parents, who were sent a letter and given the opportunity to object to the participation of their child.

## Author Contributions

AS conceived the study, analyses, and writing of the manuscript. All authors participated in the initial interpretation of findings as well as read and approved the final manuscript.

## Conflict of Interest

The authors declare that the research was conducted in the absence of any commercial or financial relationships that could be construed as a potential conflict of interest.
