# Supplementary material for: The Network Structure of Personality Pathology in Adolescence With the 100-Item Personality Inventory for DSM-5 Short-Form (PID-5-SF)
Source: Front Psychol. 2020 May 5;11:823. doi: 10.3389/fpsyg.2020.00823 (PMC7214786; doi:10.3389/fpsyg.2020.00823)
Supplement: Supplementary file 1 [file Data_Sheet_1.docx]

**Supplementary Material - Confirmatory Factor Analyses (CFAs)**

***Statistical Analyses***

The item-to-facet structure of the Dutch PID-5-SF among adolescents were examined with CFAs using Maximum Likelihood Robust (MLR) estimation in Mplus 7 (Muthén & Muthén, 2012). Specifically, the aim was to test the data fit of a factor structure that consists of 98 items that are linked to 25 facets, without allowing any residual correlations (refer to Table 1 for facet labels).

In the next steps, measurement invariance across gender groups was inspected. Assessing measurement invariance consists of several steps, with each step representing a stronger test of invariance compared to the previous step (Vandenberg & Lance, 2000). Configural invariance is the first step to establish measurement invariance, which needs to be satisfied for subsequent tests to be meaningful (Vandenberg & Lance, 2000). If the basic model factor structure (the number of factors) is equal for both groups, this signifies that the pattern of fixed and free factor loadings (and other parameters) are the same across groups, without equality constraints. To test for configural invariance, we conducted separate CFAs with the same factor structure for each group. Next, we tested for metric invariance. Metric invariance is established if the factor loadings do not significantly differ across groups. Metric invariance is tested by constraining all loadings to be equal for both groups (Vandenberg & Lance, 2000). We established metric invariance by comparing models whereby the factor loadings were free to vary for girls and boys to models whereby all factor loadings were constrained to be equal for both groups.

To examine model fit, we inspected absolute [Root Mean Square Error of Approximation (RMSEAs)] and relative fit indices [Comparative Fit Index (CFI)]. Model fit is considered acceptable should CFIs be ≥.90 and RMSEAs ≤.08 (Kline, 2005). Additionally, three commonly used criteria were considered to compare nested models. The scaled χ² - difference test should be non-significant. Also, the delta (∆) CFI and delta (∆) RMSEA should be <.010 and <.015, respectively (Chen, 2007).

***Results***

*Item-to-facet CFAs*. The fit of the item-to-facet structure was acceptable. Similarly, separate models for girls and boys had close to acceptable fit, indicating configural invariance. Fit for metric invariance models with factor constraints (i.e., metric invariance equal factor loadings) were just as good as the fit of models whereby factor loadings were not constrained (i.e., metric invariance no constraints model) for girls and boys (∆CFI < .010 and ∆RMSEA < .015 for the item-to-facet structure; though χ²-difference tests were significant, p < .05). Despite the significant χ²-difference tests, ∆CFI, and ∆RMSEA indices suggested that factor loadings were roughly equal for boys and girls, indicating metric invariance. Table S1 contains all of these model fit indices.

*Correlated residuals*. We correlated residuals between items belonging to the same facet (Table S1b; a full list of the PID-5-SF items is available at the end of this Supplementary Material document). We are aware that correlated residuals are often used in CFAs to spuriously improve model fit (Cole, Ciesla, & Steiger, 2007), and acknowledge that this may have adverse effects, as a good fitting model is not necessarily a “correct” model. However, it is known that correlated residuals may arise from several sources, including measurement method [(e.g. items with words and phrases that have similar or close to similar meanings, multidimensionality of the scales; refer to Bollen (1989) and Harrington (2008)]. For example, if we look at items that belong to the anxiousness (AnX) facet, they read: “I worry a lot about terrible things that might happen” (PID_24), “I’m always worrying about something” (PID_35), “I am a very anxious person” (PID_47), and “I’m always fearful or on edge about bad things that might happen” (PID_77). Notice that item PID_24 and PID_35 are worded very similarly, with common words like “worry”, except that PID_35 appears to cover a larger context for worrying. This might still lead to a higher correlation between the two items than for example between PID_35 and PID_47. Therefore, correlated residuals need to be included to account for the possible higher correlation. As strong a priori hypotheses about the structure of the correlated residuals of the items and facets would be premature, we chose which residuals to correlate based on modification indices alongside with indicator properties (e.g., similarities in item wording). A number of psychometric articles that have used CFA have also included correlated residuals, indicating the importance of such sources of residual correlation in the psychometric literature (Schweitzer, 2012).

We also ran the models without correlated residuals. Compared to the item-to-facet model with correlated residuals, the model without correlated residuals had similar significant factor loadings, but with a less acceptable model fit (CFI = .867). The finding suggested that while allowing residuals to correlate improved model fit, they did not change the nature of the underlying factors.

Table S1a
*Confirmatory Factor Analyses of the PID-5-SF item-to-facet structure*

|  | χ² | df | CFI | RMSEA (90% CI) | SRMR |
| --- | --- | --- | --- | --- | --- |
| Item-to-facet structure  (98 items to 25 facets) | 10428.932*** | 4283 | .900 | .027 (.027-.028) | .046 |
|  |  |  |  |  |  |
| *Configural invariance* |  |  |  |  |  |
| Boys | 7359.561*** | 4307 | .886 | .028 (.027-.029) | .047 |
| Girls | 7953.348*** | 4307 | .895 | .029 (.028-.030) | .049 |
|  |  |  |  |  |  |
| *Metric invariance* |  |  |  |  |  |
| Metric invariance no constraints | 15455.930*** | 8650 | .889 | .028 (.028-.029) | .049 |
| Metric invariance equal factor loadings | 15641.431*** | 8723 | .888 | .029 (.028-.029) | .050 |
|  |  |  |  |  |  |

*Note*: χ² = Chi-Square; df = degrees of freedom; CFI = Comparative Fit Index; RMSEA = Root Mean Square Error of Approximation and 90% Confidence Interval.

*** *p* < .001.

Table S1b
*Correlated residuals for each model*

| Item-to-facet structure | Configural invariance (Boys) | Configural invariance Girls) | Metric invariance no constraints | Metric invariance equal factor loadings |
| --- | --- | --- | --- | --- |
| PID_04 WITH PID_03;  PID_22 WITH PID_10;  PID_35 WITH PID_24;  PID_36 WITH PID_14;  PID_45 WITH PID_19;  PID_47 WITH PID_35;  PID_52 WITH PID_40;  PID_53 WITH PID_33;  PID_55 WITH PID_39;  PID_57 WITH PID_17;  PID_60 WITH PID_22;  PID_68 WITH PID_34;  PID_70 WITH PID_40;  PID_70 WITH PID_52;  PID_72 WITH PID_29;  PID_76 WITH PID_23;  PID_77 WITH PID_35;  PID_78 WITH PID_57;  PID_80 WITH PID_52;  PID_80 WITH PID_70;  PID_82 WITH PID_29;  PID_89 WITH PID_14;  PID_91 WITH PID_20;  PID_93 WITH PID_60;  PID_96 WITH PID_86;  PID_98 WITH PID_50;  PID_99 WITH PID_34;  PID_05 WITH PID_02;  PID_06 WITH PID_05;  PID_31 WITH PID_25;  PID_42 WITH PID_11;  PID_45 WITH PID_25;  PID_66 WITH PID_16;  PID_74 WITH PID_30;  PID_74 WITH PID_65;  PID_98 WITH PID_18;  PID_99 WITH PID_43;  PID_20 WITH PID_04;  PID_21 WITH PID_13;  PID_25 WITH PID_19;  PID_29 WITH PID_27;  PID_41 WITH PID_32;  PID_45 WITH PID_31;  PID_55 WITH PID_28;  PID_56 WITH PID_26;  PID_56 WITH PID_51;  PID_57 WITH PID_44;  PID_58 WITH PID_53;  PID_62 WITH PID_15;  PID_64 WITH PID_42;  PID_65 WITH PID_12;  PID_66 WITH PID_21;  PID_71 WITH PID_61;  PID_81 WITH PID_61;  PID_82 WITH PID_27;  PID_83 WITH PID_26;  PID_87 WITH PID_62;  PID_88 WITH PID_32;  PID_88 WITH PID_41;  PID_91 WITH PID_04;  PID_92 WITH PID_28;  PID_95 WITH PID_53;  PID_95 WITH PID_33;  PID_92 WITH PID_55;  PID_88 WITH PID_79;  PID_82 WITH PID_72;  PID_81 WITH PID_07;  PID_79 WITH PID_32;  PID_69 WITH PID_59;  PID_66 WITH PID_13;  PID_93 WITH PID_22;  PID_81 WITH PID_71; | PID_22 WITH PID_10;  PID_35 WITH PID_24;  PID_36 WITH PID_14;  PID_42 WITH PID_11;  PID_47 WITH PID_24;  PID_47 WITH PID_35;  PID_52 WITH PID_40;  PID_53 WITH PID_33;  PID_55 WITH PID_39;  PID_68 WITH PID_34;  PID_70 WITH PID_40;  PID_70 WITH PID_52;  PID_72 WITH PID_29;  PID_74 WITH PID_30;  PID_76 WITH PID_23;  PID_78 WITH PID_57;  PID_99 WITH PID_43;  PID_98 WITH PID_18;  PID_97 WITH PID_15;  PID_94 WITH PID_50;  PID_93 WITH PID_60;  PID_92 WITH PID_28;  PID_92 WITH PID_55;  PID_86 WITH PID_76;  PID_82 WITH PID_29;  PID_80 WITH PID_40;  PID_80 WITH PID_52;  PID_79 WITH PID_32;  PID_74 WITH PID_65;  PID_66 WITH PID_16;  PID_65 WITH PID_12;  PID_62 WITH PID_15;  PID_58 WITH PID_53;  PID_56 WITH PID_26;  PID_45 WITH PID_19;  PID_45 WITH PID_31;  PID_42 WITH PID_09;  PID_39 WITH PID_28;  PID_31 WITH PID_19;  PID_31 WITH PID_25;  PID_21 WITH PID_13;  PID_20 WITH PID_03;  PID_06 WITH PID_05;  PID_04 WITH PID_03;  PID_05 WITH PID_02;  PID_98 WITH PID_50;  PID_88 WITH PID_41;  PID_79 WITH PID_41; | PID_22 WITH PID_10;  PID_35 WITH PID_24;  PID_36 WITH PID_14;  PID_42 WITH PID_11;  PID_47 WITH PID_24;  PID_47 WITH PID_35;  PID_52 WITH PID_40;  PID_53 WITH PID_33;  PID_55 WITH PID_39;  PID_68 WITH PID_34;  PID_70 WITH PID_40;  PID_70 WITH PID_52;  PID_72 WITH PID_29;  PID_74 WITH PID_30;  PID_76 WITH PID_23;  PID_78 WITH PID_57;  PID_99 WITH PID_43;  PID_98 WITH PID_18;  PID_97 WITH PID_15;  PID_94 WITH PID_50;  PID_93 WITH PID_60;  PID_92 WITH PID_28;  PID_92 WITH PID_55;  PID_86 WITH PID_76;  PID_82 WITH PID_29;  PID_80 WITH PID_40;  PID_80 WITH PID_52;  PID_79 WITH PID_32;  PID_74 WITH PID_65;  PID_66 WITH PID_16;  PID_65 WITH PID_12;  PID_62 WITH PID_15;  PID_58 WITH PID_53;  PID_56 WITH PID_26;  PID_45 WITH PID_19;  PID_45 WITH PID_31;  PID_42 WITH PID_09;  PID_39 WITH PID_28;  PID_31 WITH PID_19;  PID_31 WITH PID_25;  PID_21 WITH PID_13;  PID_20 WITH PID_03;  PID_06 WITH PID_05;  PID_04 WITH PID_03;  PID_05 WITH PID_02;  PID_98 WITH PID_50;  PID_88 WITH PID_41;  PID_79 WITH PID_41; | PID_04 WITH PID_03;  PID_22 WITH PID_10;  PID_35 WITH PID_24;  PID_36 WITH PID_14;  PID_45 WITH PID_19;  PID_52 WITH PID_40;  PID_53 WITH PID_33;  PID_55 WITH PID_39;  PID_60 WITH PID_22;  PID_68 WITH PID_34;  PID_70 WITH PID_40;  PID_70 WITH PID_52;  PID_72 WITH PID_29;  PID_76 WITH PID_23;  PID_77 WITH PID_35;  PID_78 WITH PID_57;  PID_80 WITH PID_40;  PID_80 WITH PID_52;  PID_91 WITH PID_20;  PID_93 WITH PID_60;  PID_05 WITH PID_02;  PID_98 WITH PID_50;  PID_29 WITH PID_27;  PID_42 WITH PID_11;  PID_45 WITH PID_25;  PID_74 WITH PID_30;  PID_98 WITH PID_18;  PID_99 WITH PID_43;  PID_25 WITH PID_19;  PID_74 WITH PID_65; | PID_04 WITH PID_03;  PID_22 WITH PID_10;  PID_35 WITH PID_24;  PID_36 WITH PID_14;  PID_45 WITH PID_19;  PID_52 WITH PID_40;  PID_53 WITH PID_33;  PID_55 WITH PID_39;  PID_60 WITH PID_22;  PID_68 WITH PID_34;  PID_70 WITH PID_40;  PID_70 WITH PID_52;  PID_72 WITH PID_29;  PID_76 WITH PID_23;  PID_77 WITH PID_35;  PID_78 WITH PID_57;  PID_80 WITH PID_40;  PID_80 WITH PID_52;  PID_91 WITH PID_20;  PID_93 WITH PID_60;  PID_05 WITH PID_02;  PID_98 WITH PID_50;  PID_29 WITH PID_27;  PID_42 WITH PID_11;  PID_45 WITH PID_25;  PID_74 WITH PID_30;  PID_98 WITH PID_18;  PID_99 WITH PID_43;  PID_25 WITH PID_19;  PID_74 WITH PID_65; |

**Supplementary Material – Network Analysis**

***Robustness checks***

1) Stability and robustness of the edge weight estimates by drawing bootstrapped 95% confidence intervals (CIs); if 1000 different subsamples within the larger sample have estimates that do not change too much (i.e., narrower CIs), it is likely that estimates are representative for current sample (Figure S1).

2) Robustness of the centrality measures by generating robustness coefficients, with values above >.50 considered robust (Epskamp et al., 2017) (Figure S2).
3) Test if edges are significantly different from one another (edge weights difference test) (Figure S3).
4) Test if centrality of a node differs significantly from the centrality of another node (centrality difference test) (Figure S4).

Table S2
*Partial correlations among 25 PID-5-SF trait facets*

Table S3
*Zero-order correlations among 25 PID-5-SF trait facets*

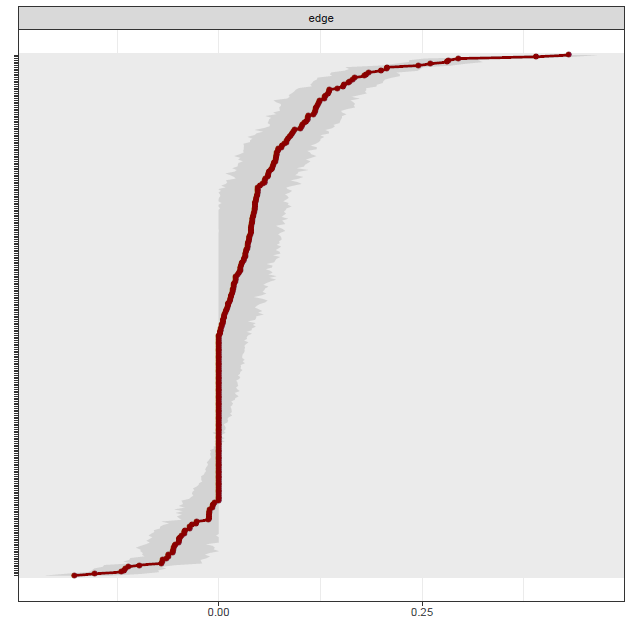


Edges

*Figure S1*. Stability of the edge weights.
*Note*: Edge weights (thick line) and the 95% CIs around these edge weights (grey bars). The edges are arranged such that the one with the highest edge-weight is at the top and the lowest edge-weight at the bottom. Ideally, narrower CIs indicate a more stable and robust estimation of the edge weights.


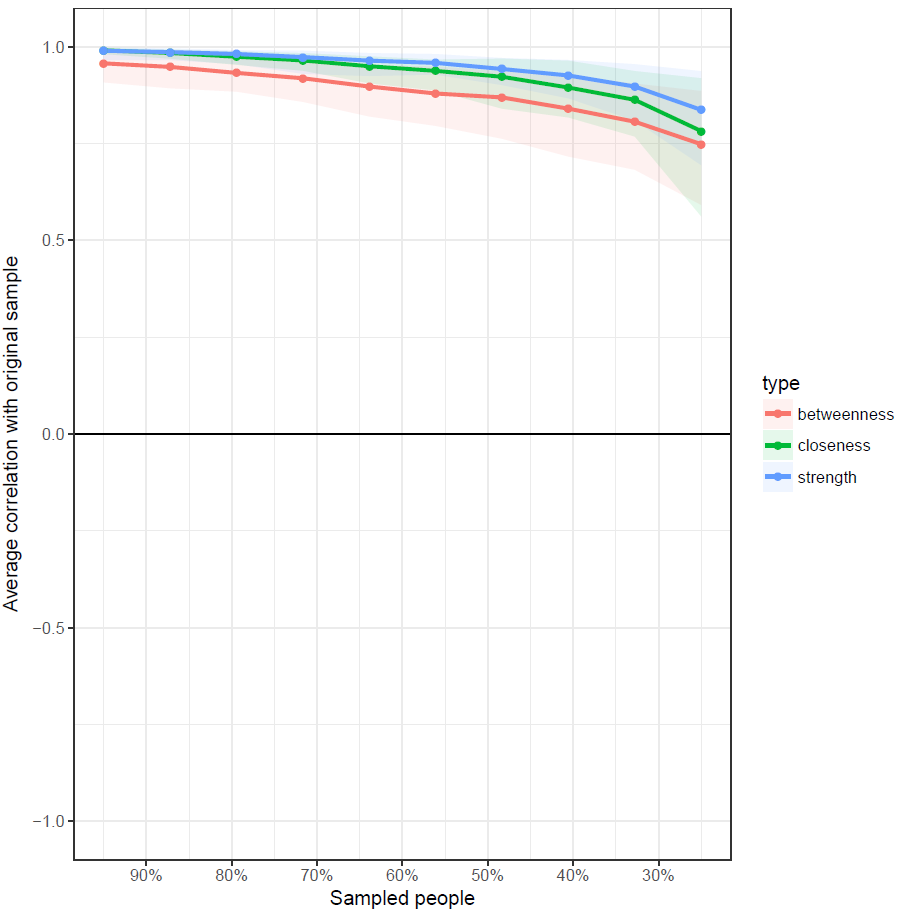


*Figure S2*. Robustness of the centrality measures.
*Note*: Represents average correlations between centrality measures in the original network with the centrality of sampled networks whereby participants are dropped. A strong correlation after dropping a high percentage of participants indicate that centrality measures in the original network can be considered robust. Generated robustness coefficients indicated that strength centrality was the most robust centrality estimate, followed by closeness, then betweenness (Figure 4). The robustness coefficients for the centrality estimates (strength: .75; closeness: .67; betweenness: .67) were > .5. As such, the centrality measures can be considered robust. Strength centrality was the most robust centrality measure. We limited the interpretation of centrality to strength centrality as strength centrality was the most robust and due to high inter-correlations among the centrality measures.

*
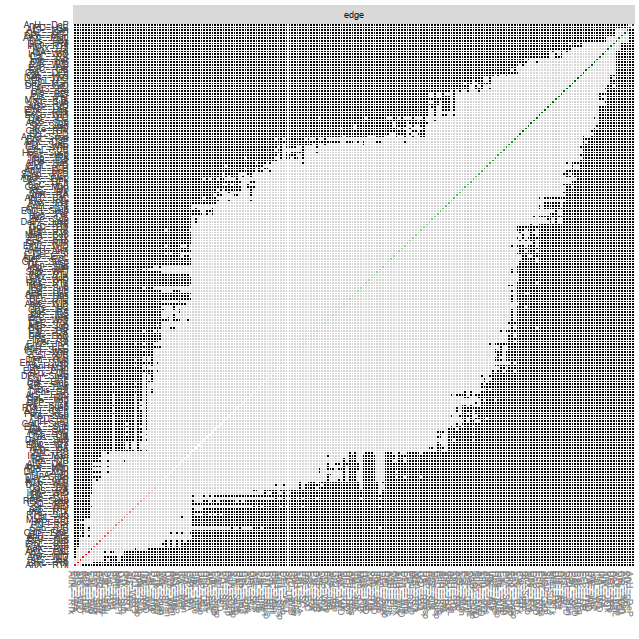
*

*Figure S3.* Edge Weights Difference Test of 25 PID-5-SF facets.
*Note*: This figure represents the edge weights of Figure 2 (diagonally colored squares). Black square indicate significant differences among edges. The edge weights difference test revealed that the two strongest edges were significantly different from the majority of the less strong edges, providing additional evidence for the robustness of the estimated network. For example, the strongest edge (Depression─Anhedonia, top right diagonal colored square) differs significantly from all other edges except for the edge below it (i.e., all squares are colored black except for the one below it). This test does not control for multiple testing.

*
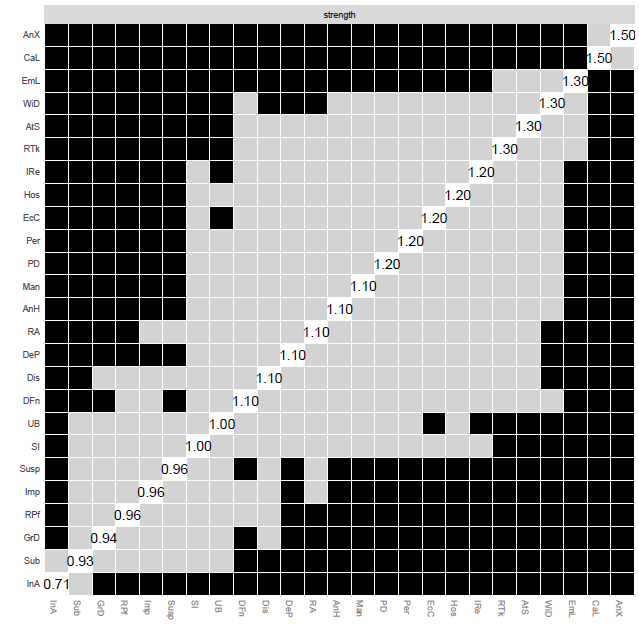
*

*Figure S4.* Strength Centrality Difference Test of 25 PID-5-SF facets.
*Note.* This graph represents whether strength centrality estimates in Figure S2 (diagonal white squares) differ significantly from each other. Black square indicate significant differences among edges. This test does not control for multiple testing.

*
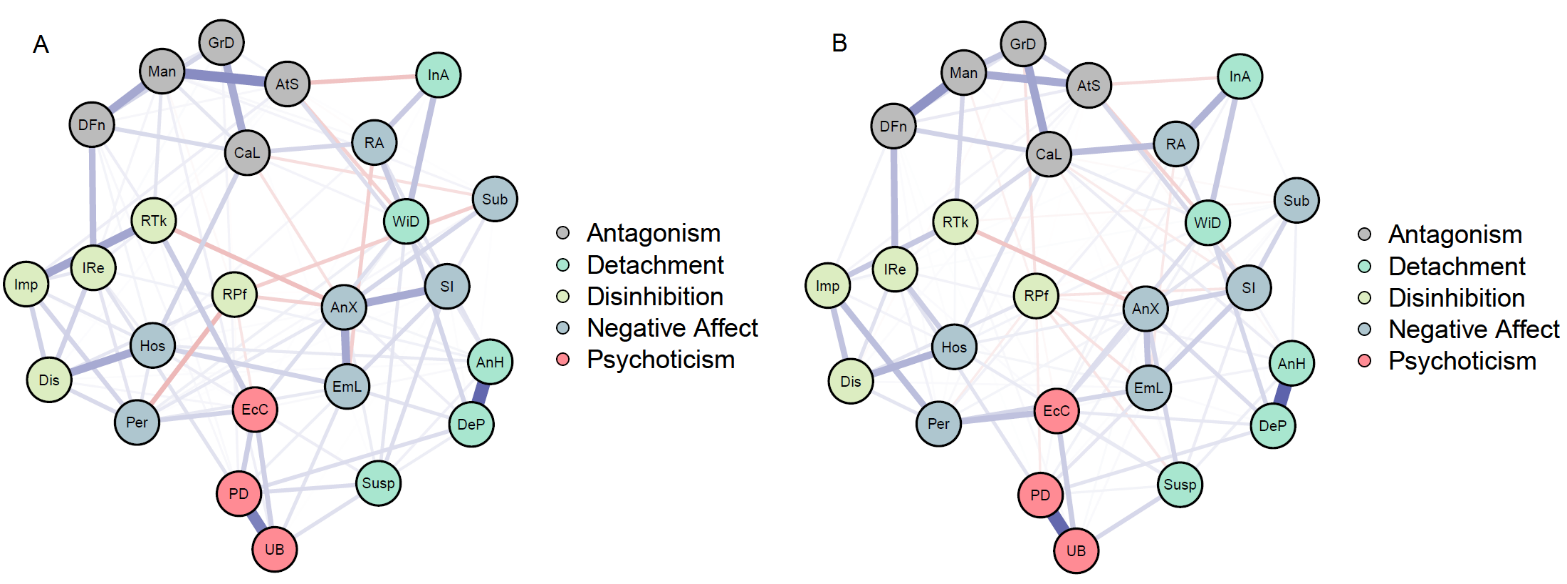
*

*Figure S5*. Network of the 25 PID-100 trait facets for girls (A) and boys (B).


*
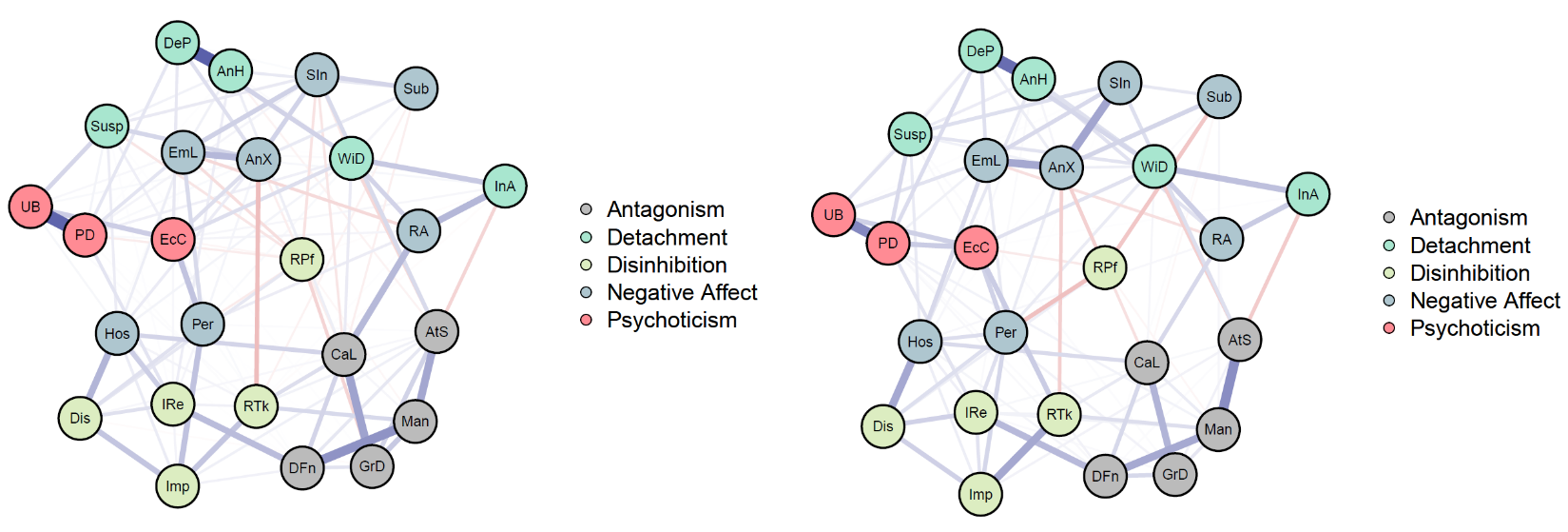
*

*Figure S6*. Network of the 25 PID-5-SF facets for random split 1 (left) and split 2 (right).

References

Bollen, K. A. (1989). Structural equations with latent variables. New York, NY: Wiley.

Chen, F. F. (2007). Sensitivity of goodness of fit indexes to lack of measurement invariance. Structural Equation Modeling, 14, 464–504. <http://dx.doi.org/10.1080/10705510701301834>

Cole, D. A., Ciesla, J. A., & Steiger, J. H. (2007). The insidious effects of failing to include design-driven correlated residuals in latent-variable covariance structure analysis. *Psychological Methods, 12*, 381–398. <https://doi.org/10.1037/1082-989X.12.4.381>

Epskamp, S., Borsboom, D., & Fried, E. I. (2017). Estimating psychological networks and their accuracy: A tutorial paper. Behavior Research Methods, 1–18. https://doi.org/10.3758/s13428-017-0862-1

Harrington, D. (2008). Confirmatory Factor Analysis. Oxford, UK: Oxford University Press.

Kline, R. B. (2005). Principles and practice of structural equation modeling (2nd ed.). New York, NY: Guilford Press.

Muthén, L. K.., & Muthén, B. O. (2007). Mplus User's Guide (6th ed.). Los Angeles, CA: Muthén & Muthén.

Schweizer, K. (2012). On correlated errors [Editorial]. European Journal of Psychological Assessment, 28(1), 1–2. https://doi.org/10.1027/1015-5759/a000094

Vandenberg, R. J., & Lance, C. E. (2000). A review and synthesis of the measurement invariance literature: Suggestions, practices, and recommendations for organizational research. Organizational Research Methods, 3, 4–69. https://doi.org/10.1177/109442810031002

|  | **The Personality Inventory for DSM-5 (PID-5)** | | | | |
| --- | --- | --- | --- | --- | --- |
|  |  | | | | |
|  | You & a description of you. This is a list of things different people might say about themselves. We are interested in how you would describe yourself. There are no right or wrong answers. So you can describe yourself as honestly as possible, we will keep your responses confidential. We’d like you to take your time and read each statement carefully, selecting the response that best describes you. | | | | |
|  |  | Very False or Often False | Sometimes or Somewhat False | Sometimes or Somewhat True | Very True or Often True |
|  |  | 0 | 1 | 2 | 3 |
| 1 | Plenty of people are out to get me. |  |  |  |  |
| 2 | I feel like I act totally on impulse. |  |  |  |  |
| 3 | I change what I do depending on what others want. |  |  |  |  |
| 4 | I usually do what others think I should do. |  |  |  |  |
| 5 | I usually do things on impulse without thinking about what might happen as a result. |  |  |  |  |
| 6 | Even though I know better, I can’t stop making rash decisions. |  |  |  |  |
| 7 | I really don’t care if I make other people suffer. |  |  |  |  |
| 8 | I always do things on the spur of the moment. |  |  |  |  |
| 9 | Nothing seems to interest me very much. |  |  |  |  |
| 10 | People have told me that I think about things in a really strange way. |  |  |  |  |
| 11 | I almost never enjoy life. |  |  |  |  |
| 12 | I am easily angered. |  |  |  |  |
| 13 | I have no limits when it comes to doing dangerous things. |  |  |  |  |
| 14 | To be honest, I’m just more important than other people. |  |  |  |  |
| 15 | It’s weird, but sometimes ordinary objects seem to be a different shape than usual. |  |  |  |  |
| 16 | I do a lot of things that others consider risky. |  |  |  |  |
| 17 | I worry a lot about being alone. |  |  |  |  |
| 18 | I often make up things about myself to help me get what I want. |  |  |  |  |
| 19 | I keep approaching things the same way, even when it isn’t working. |  |  |  |  |
| 20 | I do what other people tell me to do. |  |  |  |  |
| 21 | I like to take risks. |  |  |  |  |
| 22 | Others seem to think I’m quite odd or unusual. |  |  |  |  |
| 23 | I love getting the attention of other people. |  |  |  |  |
| 24 | I worry a lot about terrible things that might happen. |  |  |  |  |
| 25 | I have trouble changing how I’m doing something even if what I’m doing isn’t going well. |  |  |  |  |
| 26 | I keep my distance from people. |  |  |  |  |
| 27 | I don’t get emotional. |  |  |  |  |
| 28 | I prefer to keep romance out of my life. |  |  |  |  |
| 29 | I don’t show emotions strongly. |  |  |  |  |
| 30 | I have a very short temper. |  |  |  |  |

|  |  | Very False or Often False | Sometimes or Somewhat False | Sometimes or Somewhat True | Very True or Often True |
| --- | --- | --- | --- | --- | --- |
| 31 | I get fixated on certain things and can’t stop. |  |  |  |  |
| 32 | If something I do isn’t absolutely perfect, it’s simply not acceptable. |  |  |  |  |
| 33 | I often have unusual experiences, such as sensing the presence of someone who isn’t actually there. |  |  |  |  |
| 34 | I’m good at making people do what I want them to do. |  |  |  |  |
| 35 | I’m always worrying about something. |  |  |  |  |
| 36 | I’m better than almost everyone else. |  |  |  |  |
| 37 | I’m always on my guard for someone trying to trick or harm me. |  |  |  |  |
| 38 | I have trouble keeping my mind focused on what needs to be done. |  |  |  |  |
| 39 | I’m just not very interested in having sexual relationships. |  |  |  |  |
| 40 | I get emotional easily, often for very little reason. |  |  |  |  |
| 41 | Even though it drives other people crazy, I insist on absolute perfection in everything I do. |  |  |  |  |
| 42 | I almost never feel happy about my day-to-day activities. |  |  |  |  |
| 43 | Sweet-talking others helps me get what I want. |  |  |  |  |
| 44 | I fear being alone in life more than anything else. |  |  |  |  |
| 45 | I get stuck on one way of doing things, even when it’s clear it won’t work. |  |  |  |  |
| 46 | I’m often pretty careless with my own and others’ things. |  |  |  |  |
| 47 | I am a very anxious person. |  |  |  |  |
| 48 | I am easily distracted. |  |  |  |  |
| 49 | It seems like I’m always getting a “raw deal” from others. |  |  |  |  |
| 50 | I don’t hesitate to cheat if it gets me ahead. |  |  |  |  |
| 51 | I don’t like spending time with others. |  |  |  |  |
| 52 | I never know where my emotions will go from moment to moment. |  |  |  |  |
| 53 | I have seen things that weren’t really there. |  |  |  |  |
| 54 | I can’t focus on things for very long. |  |  |  |  |
| 55 | I steer clear of romantic relationships. |  |  |  |  |
| 56 | I’m not interested in making friends. |  |  |  |  |
| 57 | I’ll do just about anything to keep someone from abandoning me. |  |  |  |  |
| 58 | Sometimes I can influence other people just by sending my thoughts to them. |  |  |  |  |
| 59 | Life looks pretty bleak to me. |  |  |  |  |
| 60 | I think about things in odd ways that don’t make sense to most people. |  |  |  |  |
| 61 | I don’t care if my actions hurt others. |  |  |  |  |
| 62 | Sometimes I feel “controlled” by thoughts that belong to someone else. |  |  |  |  |
| 63 | I make promises that I don’t really intend to keep. |  |  |  |  |
| 64 | Nothing seems to make me feel good. |  |  |  |  |
| 65 | I get irritated easily by all sorts of things. |  |  |  |  |
| 66 | I do what I want regardless of how unsafe it might be. |  |  |  |  |
| 67 | I often forget to pay my bills. |  |  |  |  |
| 68 | I’m good at conning people. |  |  |  |  |

|  |  | Very False or Often False | Sometimes or Somewhat False | Sometimes or Somewhat True | Very True or Often True |
| --- | --- | --- | --- | --- | --- |
| 69 | Everything seems pointless to me. |  |  |  |  |
| 70 | I get emotional over every little thing. |  |  |  |  |
| 71 | It’s no big deal if I hurt other peoples’ feelings. |  |  |  |  |
| 72 | I never show emotions to others. |  |  |  |  |
| 73 | I’m useless as a person. |  |  |  |  |
| 74 | I’m usually pretty hostile. |  |  |  |  |
| 75 | I’ve skipped town to avoid responsibilities. |  |  |  |  |
| 76 | I like being a person who gets noticed. |  |  |  |  |
| 77 | I’m always fearful or on edge about bad things that might happen. |  |  |  |  |
| 78 | I never want to be alone. |  |  |  |  |
| 79 | I keep trying to make things perfect, even when I’ve gotten them as good as they’re likely to get. |  |  |  |  |
| 80 | My emotions are unpredictable. |  |  |  |  |
| 81 | I don’t care about other peoples’ problems. |  |  |  |  |
| 82 | I don’t react much to things that seem to make others emotional. |  |  |  |  |
| 83 | I avoid social events. |  |  |  |  |
| 84 | I deserve special treatment. |  |  |  |  |
| 85 | I suspect even my so-called “friends” betray me a lot. |  |  |  |  |
| 86 | I crave attention. |  |  |  |  |
| 87 | Sometimes I think someone else is removing thoughts from my head. |  |  |  |  |
| 88 | I simply won’t put up with things being out of their proper places. * |  |  |  |  |
| 89 | I often have to deal with people who are less important than me. |  |  |  |  |
| 90 | I get pulled off-task by even minor distractions. |  |  |  |  |
| 91 | I try to do what others want me to do. |  |  |  |  |
| 92 | I prefer being alone to having a close romantic partner. |  |  |  |  |
| 93 | I often have thoughts that make sense to me but that other people say are strange. |  |  |  |  |
| 94 | I use people to get what I want. |  |  |  |  |
| 95 | I’ve had some really weird experiences that are very difficult to explain. |  |  |  |  |
| 96 | I like to draw attention to myself. |  |  |  |  |
| 97 | Things around me often feel unreal, or more real than usual. |  |  |  |  |
| 98 | I’ll stretch the truth if it’s to my advantage. |  |  |  |  |
| 99 | It’s easy for me to take advantage of others. |  |  |  |  |
