# Supplementary material for: The Network Structure of Personality Pathology in Adolescence With the 100-Item Personality Inventory for DSM-5 Short-Form (PID-5-SF)
Source: Front Psychol. 2020 May 5;11:823. doi: 10.3389/fpsyg.2020.00823 (PMC7214786; doi:10.3389/fpsyg.2020.00823)
Supplement: Supplementary file 2 [file Data_Sheet_2.docx]

Supplementary Materials (R Code)

Uncovering the Structure of Personality Pathology in Adolescence: Complementing Factor Analyses with the Network Approach for the 100-item Personality Inventory for DSM-5 (PID-100)

1) Network of 25 PID-100 personality facets in full sample, and separately for girls and boys (both for colored and grey-scale figures)

2) Centrality measures

3) Network robustness and centrality stability

4) Compare networks of girls and boys

### 1. Load packages

library(foreign)

library(qgraph)

library(bootnet)

library(parcor)

library(Matrix)

library(psych)

library(dplyr)

library(ggplot2)

library(devtools)

library(NetworkComparisonTest)

library(huge)

##### 1 DATA

## Read in spss data file (note: change file path to suit where the file is located)

# full data

data_full<-read.spss("25facetsforR.sav", to.data.frame=TRUE)

# girls only data

data_G<-read.spss("25facetsGforR.sav", to.data.frame=TRUE)

# boys only data

data_B<-read.spss("25facetsBforR.sav", to.data.frame=TRUE)

# Create data frame of variables that we need for analysis:

# all 25 facets

data_facets<-as.data.frame(data_full[,c(1:25)]) # full data for analysis

data_girls<-as.data.frame(data_G[,c(1:25)]) # girls only

data_boys<-as.data.frame(data_B[,c(1:25)])# boys only

### Transform to normal data

data_facetsN <- as.data.frame(huge.npn(data_facets))

data_girlsN <- as.data.frame(huge.npn(data_girls))

data_boysN <- as.data.frame(huge.npn(data_boys))

##Create facet names & domain groups for graph

names<-c("AnH","AnX", "AtS", "CaL", "DFn", "DeP", "Dis", "EcC", "EmL", "GrD", "Hos", "Imp", "InA", "IRe", "Man", "PD", "Per", "RA", "RPf", "RTk", "SIn", "Sub", "Susp", "UB", "WiD")

groups<-c("Detachment","Negative Affect","Antagonism","Antagonism","Antagonism","Detachment","Disinhibition","Psychoticism","Negative Affect","Antagonism","Negative Affect","Disinhibition","Detachment","Disinhibition","Antagonism","Psychoticism","Negative Affect","Negative Affect","Disinhibition","Disinhibition","Negative Affect","Negative Affect","Detachment","Psychoticism","Detachment")

##### 3. Network : 25 PID-100 trait facets

### Coloured figures ###

g1 <- estimateNetwork(data_facetsN, default="EBICglasso") # tuning parameter default = 0.5

g2 <- estimateNetwork(data_girlsN, default="EBICglasso")

g3 <- estimateNetwork(data_boysN, default="EBICglasso")

plot(g1, layout="spring", cut=0, labels=names, directed=FALSE, legend=TRUE, vsize=5.5, maximum=.45, posCol = c('#545AA7'), border.width=2, border.color="black", minimum=.03, groups=groups, color=c('#bbbbb','#a8e6cf', '#dcedc1', '#aec6cf', '#ff8b94'))

plot(g2, layout="spring", cut=0, labels=names, directed=FALSE, legend=TRUE, vsize=5.5, maximum=.45, posCol = c('#545AA7'), border.width=2, border.color="black", minimum=.03, groups=groups, color=c('#bbbbb','#a8e6cf', '#dcedc1', '#aec6cf', '#ff8b94'))

plot(g3, layout="spring", cut=0, labels=names, directed=FALSE, legend=TRUE, vsize=5.5, maximum=.45, posCol = c('#545AA7'), border.width=2, border.color="black", minimum=.03, groups=groups, color=c('#bbbbb','#a8e6cf', '#dcedc1', '#aec6cf', '#ff8b94'))

g1mat <- getWmat(g1) # adjacency matrix

g2mat <- getWmat(g2)

g3mat <- getWmat(g3)

L <- averageLayout(g1mat, g2mat, g3mat)

Fig1a<-plot(g1, layout=L, cut=0, labels=names, directed=FALSE, legend=TRUE, vsize=5.5, maximum=.45, posCol = c('#545AA7'),border.width=2, border.color="black", minimum=.03, groups=groups, color=c('#bbbbbb','#a8e6cf', '#dcedc1', '#aec6cf', '#ff8b94'))

pdf("Fig1a.pdf", height=5, width=6.5)

plot(Fig1a)

dev.off()

Fig1b<-plot(g2, layout=L, cut=0, labels=names, directed=FALSE, legend=TRUE, vsize=5.5, maximum=.45, posCol = c('#545AA7'),border.width=2, border.color="black", minimum=.03, groups=groups, color=c('#bbbbbb','#a8e6cf', '#dcedc1', '#aec6cf', '#ff8b94'))

pdf("Fig1b.pdf", height=5, width=6.5)

plot(Fig1b)

dev.off()

Fig1c<-plot(g3, layout=L, cut=0, labels=names, directed=FALSE, legend=TRUE, vsize=5.5, maximum=.45, posCol = c('#545AA7'),border.width=2, border.color="black", minimum=.03, groups=groups, color=c('#bbbbbb','#a8e6cf', '#dcedc1', '#aec6cf', '#ff8b94'))

pdf("Fig1c.pdf", height=5, width=6.5)

plot(Fig1c)

dev.off()

### Grey scale figures ###

g1 <- estimateNetwork(data_facetsN, default="EBICglasso") # tuning parameter default = 0.5

g2 <- estimateNetwork(data_girlsN, default="EBICglasso")

g3 <- estimateNetwork(data_boysN, default="EBICglasso")

plot(g1, layout="spring", cut=0, labels=names, directed=FALSE, legend=TRUE, vsize=5.5, maximum=.45,

border.width=1, border.color="black", minimum=.03, groups=groups, theme = 'gray', negDashed=TRUE)

plot(g2, layout="spring", cut=0, labels=names, directed=FALSE, legend=TRUE, vsize=5.5, maximum=.45,

border.width=2, border.color="black", minimum=.03, groups=groups, theme = 'gray', negDashed=TRUE)

plot(g3, layout="spring", cut=0, labels=names, directed=FALSE, legend=TRUE, vsize=5.5, maximum=.45,

border.width=2, border.color="black", minimum=.03, groups=groups, theme = 'gray', negDashed=TRUE)

g1mat <- getWmat(g1) # adjacency matrix

g2mat <- getWmat(g2)

g3mat <- getWmat(g3)

L <- averageLayout(g1mat, g2mat, g3mat)

Fig1aBW<-plot(g1, layout=L, cut=0, labels=names, directed=FALSE, legend=TRUE, vsize=5.5, maximum=.45,

border.width=2, border.color="black", minimum=.03, groups=groups, theme = 'gray', negDashed =TRUE)

pdf("Fig1aBW.pdf", height=5, width=6.5)

plot(Fig1aBW)

dev.off()

Fig1bBW<-plot(g2, layout=L, cut=0, labels=names, directed=FALSE, legend=TRUE, vsize=5.5, maximum=.45,

border.width=2, border.color="black", minimum=.03, groups=groups, theme = 'gray', negDashed=TRUE)

pdf("Fig1bBW.pdf", height=5, width=6.5)

plot(Fig1bBW)

dev.off()

Fig1cBW<-plot(g3, layout=L, cut=0, labels=names, directed=FALSE, legend=TRUE, vsize=5.5, maximum=.45,

border.width=2, border.color="black", minimum=.03, groups=groups, theme = 'gray', negDashed=TRUE)

pdf("Fig1cBW.pdf", height=5, width=6.5)

plot(Fig1cBW)

dev.off()

#### Centrality measures ###

centRes <- centrality(Fig1a)

centRes$OutDegree # node strength centrality / degree centrality

centRes$Closeness

centRes$Betweenness

cor(centRes$OutDegree, centRes$Closeness, method='spearman') #0.73

cor(centRes$OutDegree, centRes$Betweenness, method='spearman') #0.88

cor(centRes$Betweenness, centRes$Closeness, method='spearman') #0.82

pdf("centplot.pdf")

centralityPlot(Fig1a) # plot centrality indices in convenient way, allow for comparison.

dev.off()

### Network robustness

## Edge Weights

boot1 <- bootnet(g1, nBoots = 1000, nCores = 4)

save(boot1, file = "boot1.Rdata")

load(file = "boot1.Rdata")

pdf("boot1.pdf")

plot(boot1, labels = FALSE, order = "sample" )

dev.off()

## Centrality

boot2 <- bootnet(g1, nBoots = 1000, type = "case", nCores = 4)

save(boot2, file = "boot2.Rdata")

load(file = "boot2.Rdata")

pdf("boot2.pdf")

plot(boot2)

dev.off()

## Centrality stability coefficient

corStability(boot2) # "CS-coefficient should not be below 0.25, and preferably above 0.5"

## betweenness closeness strength

#0.6721649 0.6721649 0.7500000

## Edge weights difference tests

# Gray boxes indicate nodes or edges that do not differ significantly from one-another

# and black boxes represent nodes or edges that do differ significantly from one-another

boot3 <- plot(boot1, "edge", plot = "difference", onlyNonZero = TRUE, order = "sample", labels=FALSE)

## Centrality nodes difference tests

boot4 <- plot(boot1, "strength", order="sample", labels=FALSE)

pdf("boot3.pdf")

plot(boot3)

dev.off()

pdf("boot4.pdf")

plot(boot4)

dev.off()

### Network comparison: boys and girls. note NCT cannot handle missing data, so we use the edited data sets

##### 1 DATA

# Read in spss data file

data1<-read.spss("25facetsGforR_edited.sav", to.data.frame=TRUE)

data2<-read.spss("25facetsBforR_edited.sav", to.data.frame=TRUE)

# Create data frame of variables that we need for analysis:

# all 25 facets

data_girls1<-as.data.frame(data1[,c(1:25)]) # girls data for analysis

data_boys1<-as.data.frame(data2[,c(1:25)]) # boys data for analysis

### Transform to normal data

data_girlsN <- as.data.frame(huge.npn(data_girls1))

data_boysN <- as.data.frame(huge.npn(data_boys1))

### to resolve Error in match.names(clabs, names(xi)) error

class(data_girlsN)

class(data_boysN)

compare1<-NCT(data_girlsN, data_boysN, binary.data=FALSE, it=1000, gamma=.5, progressbar=TRUE, paired=FALSE)

save(compare1, file = "compare1.Rdata")

load(file = "compare1.Rdata")

plot(compare1, what="network") # is structure different? 0.151 p= 0.085 <- no

plot(compare1, what="strength") # is global strength different? 12.4 vs 12.3 p= 0.835 <- no
